# Molecular evolution of the VacA p55 binding domain of *Helicobacter pylori* in mestizos from a high gastric cancer region of Colombia

**DOI:** 10.7717/peerj.6634

**Published:** 2019-05-06

**Authors:** Andrés J. Gutiérrez-Escobar, María M. Bravo, Orlando Acevedo, Steffen Backert

**Affiliations:** 1 Universidad de Ciencias Aplicadas y Ambientales U.D.C.A. Doctorado en Ciencias Biológicas, Pontificia Universidad Javeriana., Bogotá, Colombia; 2 Division of Microbiology, Department of Biology, Friedrich Alexander University Erlangen/Nuremberg, Erlangen, Germany; 3 Grupo de Investigación en Biología del Cáncer, Instituto Nacional de Cancerología, Bogotá, Colombia; 4 Grupo de Biofísica y Bioquímica Estructural, Facultad de Ciencias, Pontifica Universidad Javeriana, Bogotá, Colombia

**Keywords:** Positive selection, Gene convergence, Functional divergence, *Helicobacter pylori*, VacA, Evolution

## Abstract

The stomach bacterium* Helicobacter pylori* is one of the most prevalent pathogens in humans, closely linked with serious diseases such as gastric cancer. The microbe has been associated with its host for more than 100,000 years and escorted modern humans out of Africa. *H. pylori* is predominantly transmitted within families and dispersed globally, resulting in distinct phylogeographic patterns, which can be utilized to investigate migrations and bioturbation events in human history. Latin America was affected by several human migratory waves due to the Spanish colonisation that drastically changed the genetic load and composition of the bacteria and its host. Genetic evidence indicates that independent evolutionary lines of *H. pylori* have evolved in mestizos from Colombia and other countries in the region during more than 500 years since colonisation. The vacuolating cytotoxin VacA represents a major virulence factor of the pathogen comprising two domains, p33 and p55, the latter of which is essential for binding to the host epithelial cell. The evolution of the VacA toxin in Colombia has been strongly biased due to the effects of Spanish colonization. However, the variation patterns and microevolution of the p55 domain have not yet been described for this population. In the present study, we determined the genetic polymorphisms and deviations in the neutral model of molecular evolution in the p55 domain of 101 clinical *H. pylori* isolates collected in Bogotá, a city located in Andean mountains characterized by its high gastric cancer risk and its dominant mestizo population. The microevolutionary patterns of the p55 domain were shaped by recombination, purifying and episodic diversifying positive selection. Furthermore, amino acid positions 261 and 321 in the p55 domain of VacA show a high variability among mestizos clinical subsets, suggesting that natural selection in *H. pylori* may operate differentially in patients with different gastric diseases.

## Introduction

*H. pylori* is a highly successful Gram-negative bacterium that colonizes the stomach of about 50% of the human world population (Perez-Perez, Rothenbacher & Brenner, 2004; Khalifa, Sharaf & Aziz, 2010). The infection is associated with superficial gastritis; however, a subset of individuals can develop ulcers (Ernst & Gold, 2000) or two aggressive forms of cancer, mucosa-associated lymphoid tissue (MALT) lymphoma or gastric adenocarcinoma (Parsonnet et al., 1991; Parsonnet et al., 1994; Blaser, 1998; Posselt, Backert & Wessler, 2013). Thus, *H. pylori* infection is recognized as the most important risk factor for gastric cancer development (International Agency for Research on Cancer (IARC), 1994; Helicobacter and Cancer Collaborative Group, 2001). In Colombia, gastric cancer is the fourth cause of deaths by cancer (Ferlay et al., 2013) and the risk to develop this disease increases following the altitudinal gradient (Torres et al., 2013). In this country, an inverse relation in gastric cancer risk has been observed between inhabitants from the Andes with high risk and those located at the coasts with low risk (Kodaman et al., 2014). Bogotá and its surrounding towns, the places from where the clinical isolates of the present study were obtained, are located in the Andean mountain and consequently have a high gastric cancer risk (Marion & Murillo, 2004) and its population is conformed predominantly by mestizos (Ossa et al., 2016; Adhikari et al., 2016).

*H. pylori* and humans have co-evolved for at least 100,000 years, and the bacteria have mimicked the settlement pattern of its host (Linz et al., 2000). Seven major populations of *H. pylori* have been discovered on our planet: hpEurope, hpNEAfrica, hpAfrica1, hpAfrica2, hpAsia2, hpSahul and hpEastAsia (Yamaoka, 2009; Falush et al., 2003; Achtman, 2007; Achtman et al., 1999; Moodley et al., 2009; Devi et al., 2007). About 12,000 to 15,000 years ago, humans carrying the hpEastAsia *H. pylori* population migrated to the American continent by crossing the Bering Strait, which developed towards a new hspAmerind cluster (Kersulyte et al., 2010). However, the Spanish colonization era, starting in the 15th century, changed the load of pathogens in the native human population of Latin America (Bianchine & Russo, 1992). Thus, new strains of *H. pylori* were introduced to the continent by European conquerors and also African slaves, which, through recombination, gene conversion and natural selection, produced unique evolutionary lineages of the bacterium in the mestizos of Colombia and other countries of Latin America (Gutiérrez-Escobar et al., 2017; Thorell et al., 2017).

One of the most studied virulence factors of *H. pylori* is the vacuolating cytotoxin VacA (Leunk et al., 1988; Chung et al., 2010; Backert & Tegtmeyer, 2010; Jang et al., 2010). This toxin is present in all *H. pylori* strains and does not have homologues in other bacterial species (Cover & Blanke, 2005). VacA displays two main cellular localizations: on the bacterial cell surface (Ilver et al., 2004) or as a secreted toxin of about 88 kDa (Cover & Blaser, 1992). The secreted toxin is cleaved into two smaller products, called p33 and p55. While p55 binds to the host cell and has been used to classify the m1 or m2 allelic VacA types, p33 and part of p55 are responsible for the intracellular vacuolating activity (Ye, Willhite & Blanke, 1999; Torres, McClain & Cover, 2004; Ji et al., 2000). Previous phylogenetic analysis using the full-length VacA sequence revealed three discrete phylogenetic clusters, one for non-Asian strains, other for exclusively Asiatic strains and the last conformed by a worldwide mixture, the first two were assigned to the type m1; meanwhile the last to the type m2 (Gangwer et al., 2010). In the same study, they also showed that the phylogeny of the p55 domain resembles the phylogeography of full-length VacA and found that adaptive evolution has driven its divergence patterns (Gangwer et al., 2010).

A recent study has also shown that the evolution of VacA in Latin America has been strongly biased due to the effects of Spanish colonization (Gutiérrez-Escobar et al., 2017). However, neither the phylogeny nor the way in which natural selection has operated on the p55 domain has been described yet in *H. pylori* isolates from this region. In this context, the aim of the present study was to describe the genetic diversity and microevolution of the p55 domain in a large group of clinical *H. pylori* isolates obtained from mestizos in a high gastric cancer risk zone from Colombia.

## Material and Methods

The DNA and protein sequences corresponding to the p55 domain of VacA were obtained from 101 genomes previously sequenced by our group from the *H. pylori* stock collection at the Instituto Nacional de Cancerología in Bogotá (Gutiérrez-Escobar et al., 2017). The stock collection was obtained from inhabitants of Bogota, Tunja and surrounding towns. The study region is located in a high plateau (8,660 ft), also called the Bogota savanna, and is part of the Altiplano Cundiboyacense at the Eastern Cordillera of the Andes. This region is characterized by its high gastric cancer risk (International Agency for Research on Cancer (IARC), 1994; Helicobacter and Cancer Collaborative Group, 2001). The sequences were categorised in four groups according to the gastric disease state as follows: 31 cases of gastritis (G), 17 cases of gastric adenocarcinoma (GA), 27 cases of atrophic gastritis (AG), and 26 cases of intestinal metaplasia (IM). For the m region characterization, sequences corresponding to m1, m2 and m1/m2 types were downloaded from Genbank. For m1: Q48245; chimeras (ch): Q9 kJA6, Q6DLS8; and m2: Q48253 (Cover et al., 1994; Atherton et al., 1999. The sequences were aligned using MUSCLE software (Edgar, 2004); and displayed in ESPript http://espript.ibcp.fr (Robert & Gouet, 2014).

### Population statistics, neutrality test and phylogenetic reconstruction

Basic population genetic estimators were calculated as number of haplotypes (H), haplotype diversity (Hd), nucleotide diversity (Pi), and average number of nucleotide differences (k) using DnaSP v5.10 software (Librado & Rozas, 2009). The deviation of neutral expectations was calculated applying the Tajima test (Tajima, 1989) in MEGA v7 (Caspermeyer, 2016). The 101 p55 VacA sequences from Colombian isolates were aligned using MUSCLE (Edgar, 2004), then the evolutionary model and phylogeny were determined using MEGA v7 (Caspermeyer, 2016) applying the NJ algorithm (Huelsenbeck & Ronquist, 2001) and 1,000 bootstrap repetitions for statistical robustness.

### Recombination and gene conversion analysis

The p55 *vacA* sequences were analyzed in overall average and per disease group. Gene conversion was tested using the Betran’s method (Betran et al., 1997) and recombination using the Rm estimator or the minimum number of recombination events (Hudson & Kaplan, 1985) implemented in the software DnaSP v5.10 (Librado & Rozas, 2009).

### Type I functional divergence and positive selection analyses

I-TASSER was used to predict the structure of p55 VacA from Colombian samples (Yang et al., 2015) using 2QV3 as template (Gangwer et al., 2007). The root mean square deviation (RMSD), superimposition and surface protein analyses were performed using Chimera v1.11.2 (Pettersen et al., 2004). DIVERGE v3 (Gu, 1999; Gu et al., 2013) was used to estimate type I functional divergence, which detects functional changes in a protein based on site-specific shifts of evolutionary rates (Gu, 1999; Gu et al., 2013). The software tests whether a significant change in evolution rate has occurred, by calculating the coefficient of divergence (*θ*D). Positive and negative selection was evaluated as the proportions of synonymous to non-synonymous substitution rates. The p55 VacA alignments were corrected for recombination using the Single Break Point (SBP) algorithm and then, the Fixed Effects Likelihood (FEL) and the Internal Fixed Effects Likelihood (IFEL) methods were performed using the datamonkey server (Kosakovsky Pond & Frost, 2005). The episodic diversifying selection was detected using the Mixed Effects Model for Episodic Diversifying Selection (MEME) algorithm (Murrell et al., 2012) also implemented in datamonkey server. Only sites SBP corrected with a *p* < 0.05 were considered significant.

### Immune selection test and significant sequence variation between groups

Initially, the full protein sequence of p55 VacA was used to identify regions with positive scores for B cell epitopes using the server http://tools.iedb.org/bcell/ (Jespersen et al., 2017; Larsen, Lund & Nielsen, 2006). Then, positive selected sites shared by the FEL, IFEL and MEME tests were mapped against the significant epitope predicted regions. To identify significant sequence variation between sequence groups a multiple sequence alignment was performed using MUSCLE software (Edgar, 2004), then a chi-square test of independence was performed on all non-conserved columns using a *p* < 0.05 as a threshold value. The degrees of freedom were assigned according to the equation: number of observed residues at each aligned position −1 multiplied by the number of groups being calculated −1. Those columns that significantly deviated from random were subject to a Person’s chi-square to determine the pair observed groups skewing of the data; the calculations were performed using the Meta-cast tool implemented in http://www.viprbrc.org (Pickett et al., 2013).

## Results

**Figure 1:**
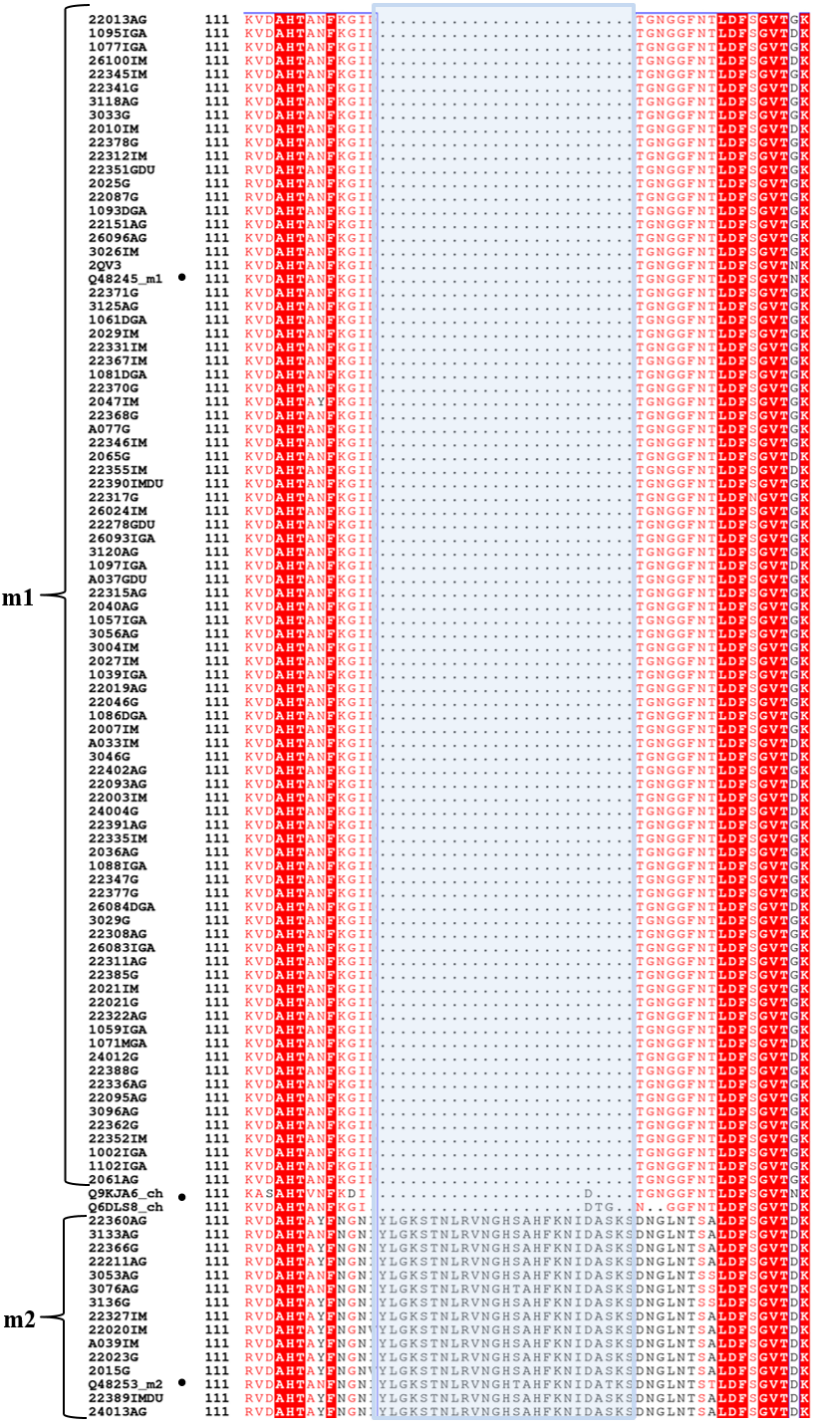
Protein sequence alignment of p55 VacA from *H. pylori* of Colombian mestizos. The strain names are given to the left. The middle section of VacA (shaded with light blue) differentiates the m1 and m2 types, respectively. The m1 sequence types, characterized by its lack of the middle section (top sequences), were the most prevalent in the studied population in comparison to the m2 sequence type, which has a clear middle section (bottom sequences). Amino acids marked with red correspond to strictly conserved residues between all aligned sequences. Black dots mark the chosen reference VacA sequences for m1, m2 and chimera (ch) alleles (Atherton et al., 1999; Cover et al., 1994). The alignment was performed using Muscle software and displayed in ESPript server.

In our previous analysis of genetic differentiation of the *vacA* gene using 101 *H. pylori* genome sequences of mestizos from a high gastric cancer region in the Andes of Colombia and 34 reference genomes showed that the Colombian alleles are more similar to that of HpEurope or HspWestAfrica, differentiating them from HspAsia and HspAmerind strains (Gutiérrez-Escobar et al., 2017). This indicates that the Colombian population may have a remarkable European-African component with regard to the VacA type, which was not yet studied. In the present study, we performed a molecular evolution analysis of the p55 domain of VacA from the 101 *H. pylori* isolates (Table S1). For this purpose, the p55 protein sequences were aligned against four reference sequences belonging to the m1, m2 and chimera allele types. The alignment revealed that 84% of the sequences from Colombian isolates belonged to the m1 type, whereas 15.2% belonged to the m2 type (Fig. 1).

**Figure 2:**
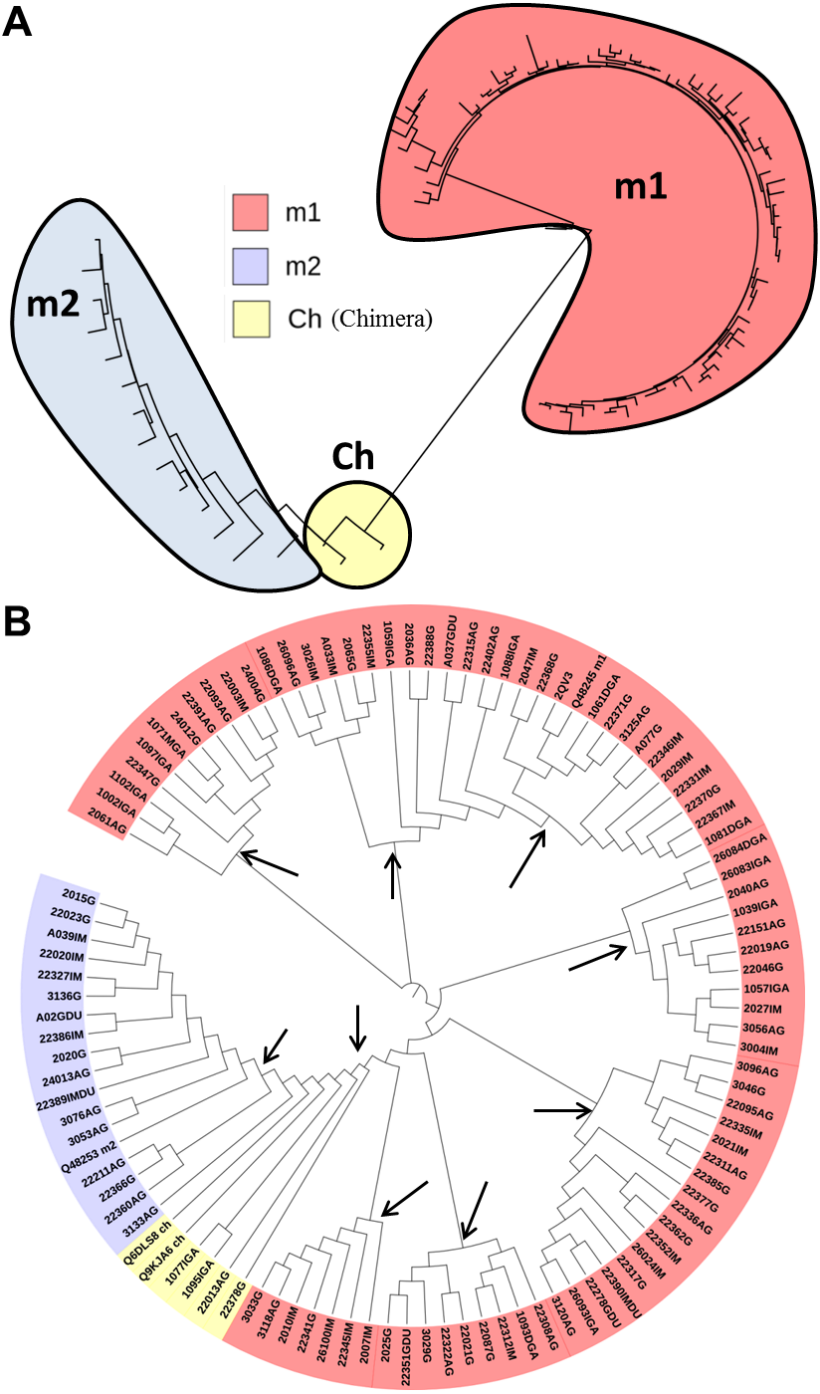
Phylogenetic tree of p55 VacA from *H. pylori* of Colombian mestizos. The tree was inferred using the Neighbor-Joining method (Saitou & Nei, 1987). (A) The tree is drawn to scale, with branch lengths in the same units as those of the evolutionary distances used to infer the phylogenetic tree. The topology robustness was calculated using the averages of 2,000 replicates from the bootstrap test (Felsenstein, 1985). Only significant consensus tree branches are shown. The evolutionary model that best fitted the alignment was the JTT+G+F+I (Jones, Taylor & Thornton, 1992) showing a BIC (Bayesian Information Criterion) = 15,807, *ln*L = −6,341,5. The rate variation among sites was modeled with a gamma distribution = 2. The analysis involved 101 amino acid sequences. All positions containing gaps and missing data were eliminated. Evolutionary analyses were conducted in MEGA7 (Kumar, Stecher & Tamura, 2016). (B) The phylogenetic tree without distances showing discrete clades (arrows) accounting for the diversification of the domain in the studied samples.

The *vacA* gene fragment that encodes for the p55 domain in Colombian clinical isolates showed 431 segregating sites and 95 haplotypes with a haplotype diversity of 0.999. The nucleotide diversity (*π*) was 0.0741 and the average number of nucleotide differences (k) was 99.523. The Tajima’s D test was 0.669, indicating that rare alleles are at low frequency in the population. The phylogenetic tree of p55 VacA showed three major clades corresponding to the m1, m2 and chimera types. Although the phylogenetic tree has very short branches indicating a very recent evolutionary history, seven independent lines were identified for the m1 sequences, one for chimeras and one for m2 sequences, suggesting that diversifying selection has influenced the evolution of this domain in the high gastric risk zone in Colombia (Fig. 2). Recombination and gene conversion played an important role in the evolution of p55 *vacA* gene sequences. The Betran method showed 24 gene conversion tracts for the entire sample. However, when the sequences were analyzed per disease group, all shared genetic tracts. The Hudson R estimator showed 98 minimal recombination events varying from the AG population with 69 events until the GA with 32 (Table 1).

**Figure 3:**
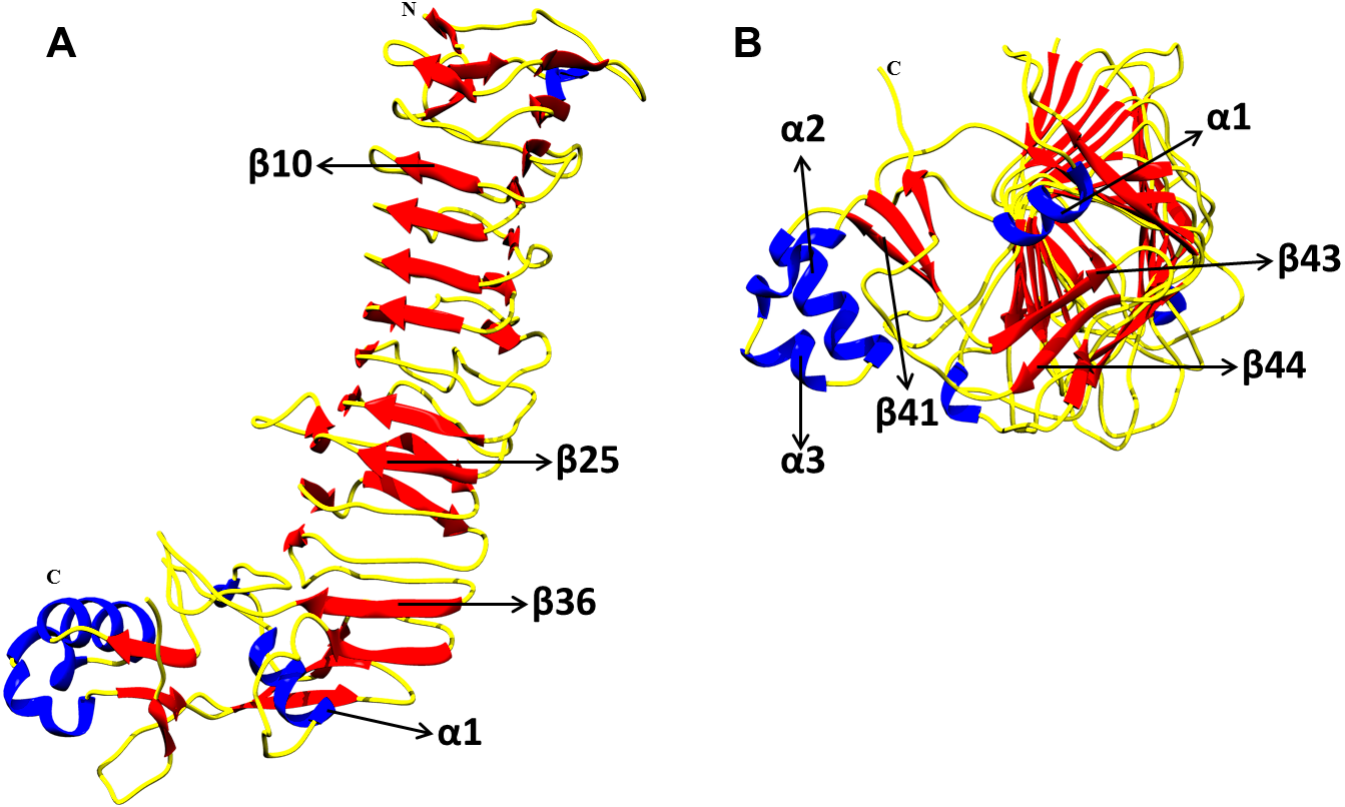
Structure model of p55 VacA from *H. pylori* of Colombian mestizos. The I-TASSER server (Yang et al., 2015) was used to predict the structure of p55 domain using the protein sequence named as 1,077 from the Colombian samples as query and the crystal structure 2QV3 as template (Gangwer et al., 2007). The root mean square deviation (RMSD), superimposition and surface protein analyses were performed using Chimera v1.11.2 (Pettersen et al., 2004). The conformation of α helixes and the β sheets between the template and the model was strongly similar as displayed. (A) Lateral view; (B) Bottom view.

As next we aimed to investigate p55 at the amino acid level. For this purpose, the p55 protein model of the Colombian sequence 1077GA was obtained by using the published crystal structure (PDB code 2QV3) as a template, which showed a high structural similarity (Gangwer et al., 2007). The N- and C-termini of the p55 VacA model showed the typical *β*-strands and *α*-helices previously identified in the 2QV3 crystal (Gangwer et al., 2007). Likewise, the RMSD predicted for the above p55 VacA model was 0.198 Å for alpha carbon, and 0.61 Å  for the backbone. About 83% of the amino acid residues were surface-exposed and 17% were classified as buried inside the protein (Figs. 3A/ 3B).

The clades previously identified in the phylogenetic tree were used to detect functional divergence events amongst the p55 VacA sequences. Although, the pairwise comparisons between the clades 1 and 2 showed that the sites at amino acids P454 and L342 have a *θ*D>0.5, this result was not significant (*p* > 0.07). The dataset was corrected for recombination using the Single Break Point (SBP) algorithm and then positive and negative selection analyses were performed. The analysis showed that according to the FEL test, 3.7% residues evolved under positive selection and 22.2% by purifying selection, and the IFEL test indicated that 3.7% evolved by positive selection and 17.3% under purifying selection (Table 2 and Table S2 to clarify the numbering system). Likewise, 8.7% of p55 VacA residues showed episodic diversifying selection according to the MEME test (Table 3 and Table S2).

**Table 1:** Recombination and gene conversion events in the VacA p55 domain sequence obtained from Colombian mestizos.

**Recombination events**			**Gene conversion**	
	**n**	**Rm**		**Tracts**
Average	101	98	G *vs*. AG	7
AG	25	69	G *vs.* IM	1
G	32	64	G *vs.* GA	4
IM	26	59	AG *vs.* IM	3
GA	17	32	AG *vs.* GA	3
			**IM***vs.***GA**	6

**Notes.**

Average: the total number of recombination events in the 101 sequences.

Rm minimum recombination events G Gastritis AG atrophic gastritis IM intestinal metaplasia GA gastric adenocarcinoma

**Table 2:** Positive selection test of VacA p55 sequences.^a^

**Amino acid position**	**dS**	**dN**	**dN/dS**	**dN-dS**	***p*-value**
N11	0	2,043	Infinite	2,135	0,015
Y21	0	4,172	Infinite	4,361	0,002
H33	0	3,374	Infinite	3,527	0,004
V39	0	2,787	Infinite	2,913	0,001
E180	0	3,081	Infinite	3,221	0,003
K206	0	2,307	Infinite	2,411	0,015
A241	0	1,811	Infinite	1,893	0,024
E246	0	1,013	Infinite	1,059	0,031
V261^*^	0	1,108	Infinite	1,158	0,025
L291	0	1,643	Infinite	1,718	0,006
R321^*^	0	3,523	Infinite	3,683	0,028
E338	0	3,881	Infinite	4,057	0,020
H399	6,8E–15	3,922	5,798E+14	4,100	0,020
G423	4,1E–01	3,204	7,726	2,916	0,017
I424	0	1,496	Infinite	1,564	0,039
Y437	1,0E–06	1,058	1,058,420	1,106	0,032
P443	1,6E–15	3,849	2,416E+15	4,024	0,001

**Notes.**

a FEL tests for positive selection of VacA p55 sequences. Shown sites were detected also using the IFEL test. The dS are synonymous sites and dN are non-synonymous sites; the dN/dS is the Omega value; and dN-dS is the normalized test. Stars indicate amino acids under positive selection that were also found to be significantly different between disease groups.

**Table 3:** Episodic diversifying positive selection test of VacA p55 sequences.^a^

Amino acid position	*α*	*β*	Pr[*β* = *β* −]	*β* +	Pr[*β* = *β* +]	*p*-value
423^*^	0,388	0,000	0,946	105,075	0,054	9,06E–09
480	0,000	0,000	0,963	243,320	0,037	1,05E–07
399	0,000	0,000	0,932	68,495	0,068	2,90E–07
481	0,000	0,000	0,960	96,483	0,040	9,33E–07
437^*^	0,000	0,000	0,982	155,811	0,018	1,35E–06
101	0,000	0,000	0,992	1,407,450	0,008	6,68E–06
388^*^	0,000	0,000	0,931	159,701	0,069	1,17E–05
443^*^	0,000	0,000	0,842	21,711	0,158	2,19E–05
478	0,000	0,000	0,908	119,998	0,092	3,31E–05
479	0,174	0,174	0,968	92,166	0,032	4,03E–05
449	0,000	0,000	0,975	68,471	0,025	6,29E–05
476	0,435	0,000	0,981	241,668	0,019	1,30E–04
89	0,000	0,000	0,974	50,406	0,026	2,70E–04
100	0,000	0,000	0,983	427,917	0,017	2,83E–04
268	0,000	0,000	0,961	27,107	0,039	4,71E–04
74	0,000	0,000	0,980	144,071	0,020	7,58E–04
21^*^	0,000	0,000	0,840	40,252	0,160	1,25E–03
246^*^	0,000	0,000	0,962	20,210	0,038	2,03E–03
39^*^	0,000	0,000	0,688	9,174	0,312	2,20E–03
274	0,000	0,000	0,977	22,648	0,023	3,00E–03

**Notes.**

a MEME tests for episodic diversifying selection of VacA p55 sequences. Stars indicate amino acid positions that were identified under positive selection by the FEL and IFEL tests as shown in Table 2. The *α* are synonymous substitutions and *β* are non-synonymous substitutions.

**Figure 4:**
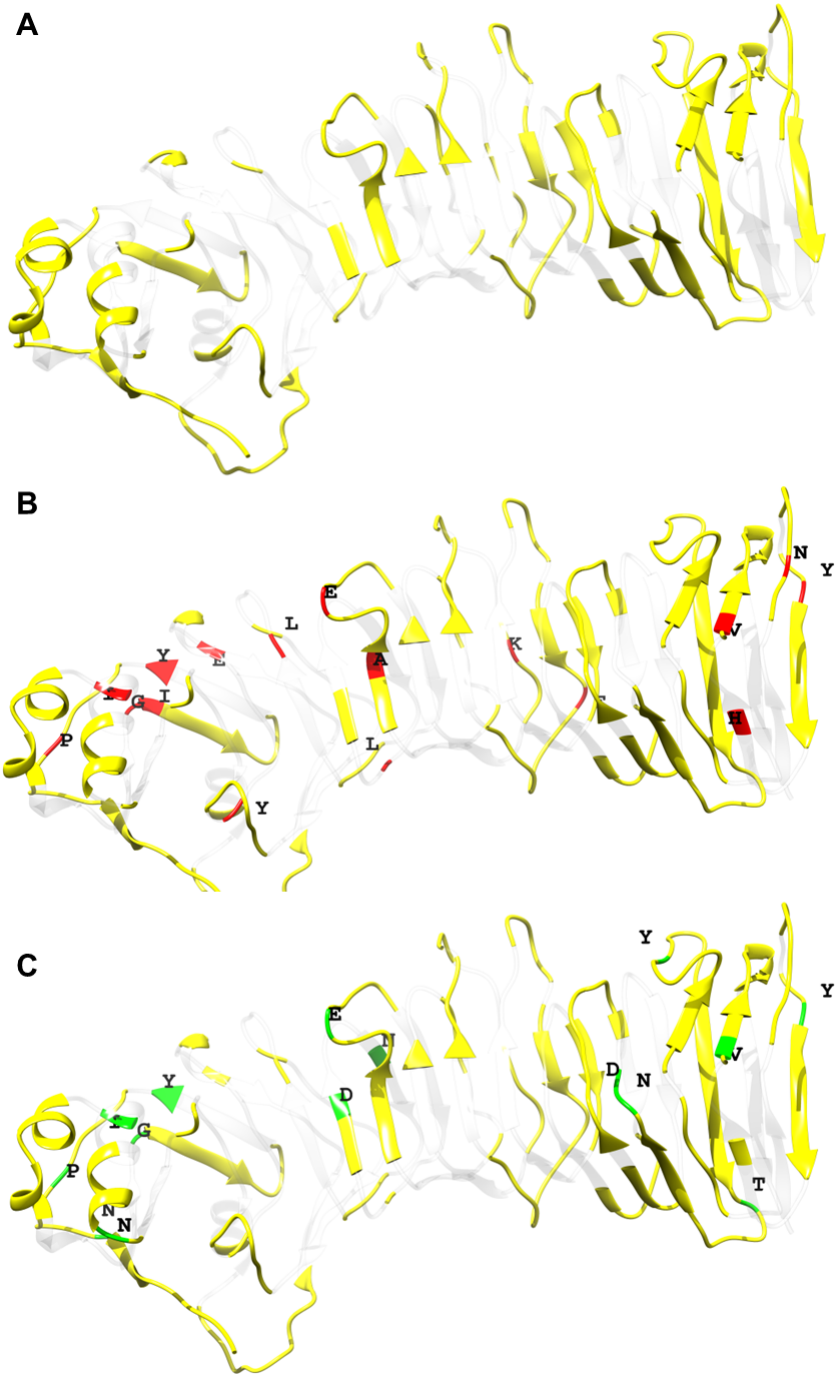
Relation between natural selection and immune recognition operating on p55 VacA from *H. pylori* of Colombian mestizos. (A) In yellow the regions predicted to be recognized by humoral immunity using the server http://tools.iedb.org/bcell/ (Jespersen et al., 2017). (B) Positive selected amino acid positions shared by the FEL, IFEL were mapped and shown in red. (C) Episodic diversified amino acid positions are indicated in green. Superimposition suggests a possible role of immune selection on the microevolution of variants for the p55 in this region of Colombian strains. Only sites with a *p* < 0.05 were shown.

To identify if immune selection also played a role in the evolution of p55 VacA, an epitope prediction test was performed. According to this prediction, the 48.3% of p55 VacA has the potential to be recognized by humoral immunity (Fig. 4A). All the positive and episodic diversifying selected sites detected using the FEL, IFEL and MEME tests were mapped against the p55 VacA structure. About 58.8% of the positive selected sites according to the FEL and IFEL test and the 35% of the episodic diversified selected sites according to MEME test were located in the regions predicted to be recognized by the host humoral immunity. This result implies that immune selection may have contributed to shaping the evolutionary pattern of this protein domain in strains from Colombia (Figs. 4B/4C). Besides, the amino acid residues R321 and V261 (see Table S2), both recognized by FEL and IFEL test under positive selection, showed significant variation between all possible pairwise combinations of the p55 VacA groups when the Pearson chi-squared test was performed (Table 4).

## Discussion

Gene diversification and duplication are important sources of biological innovation during evolution (Zhang, 2003). For example, duplicated genes evolve in two pathways; they either become functional novelties or become functionless (Lynch & Conery, 2000). It has been shown that gene duplication and frameshift mutations have an important role in *vacA* evolution, not only in *Helicobacter pylori* but also in other species of this genus (Ito et al., 1998; Foegeding et al., 2016). Likewise, secreted VacA recognizes several host cell receptors; for example, the proteins LRP1 (Yahiro et al., 2012), RPTP (Yahiro et al., 2003; Yahiro et al., 2004) and EGFR (Seto et al., 1998; Tegtmeyer et al., 2009) as well as sphingomyelin (Utt, Danielsson & Wadstrom, 2001; Gupta et al., 2008), glycosphingolipids (Gupta, Wilson & Blanke, 2010), heparan sulphate (Roche et al., 2007) and phospholipids (Molinari et al., 1998), indicating that functional novelty may have occurred during evolution.

The genetic structure of p55 VacA from Colombian isolates indicates that some alleles evolves under positive selection according to Tajima’s D (*D* = 0.669), and the high number of haplotypes suggests a low frequency of rare alleles. It is important to stress that the observed nucleotide diversity is low, indicating that a population contraction could have taken place in this region from Colombia, but advantageous variants of p55 VacA were maintained through balancing selection. Several studies have suggested that Latin American mestizos have an admixture of ancestries from Europe and Africa (Falush et al., 2003; Thorell et al., 2016). After colonization by the European conquerors, *H. pylori* may evolved alongside its mestizo host producing independent evolutionary lines as shown by multilocus sequence typing (MLST), phylogenomic and AlpA adhesin analyses using genomes sequences of the same isolates from which we obtained the p55 sequences (Gutiérrez-Escobar et al., 2017; Gutiérrez-Escobar et al., 2018). This could explain a possible population contraction and the dominant purifying selection detected by analysis of the p55 VacA domain.

**Table 4:** Significant protein sequence variation between VacA p55 from Colombia.^a^

Amino acid positions	**Chi-square**	***p*-value**	**DF**	**Residue Diversity**
R261	14,406	0,002	3	G (31 I)
				AG (22 I, 5 V)
				IM (26 I)
				GA (17 I)
L321	17,699	0,039	9	G (6 K, 19 Q, 6 R)
				AG (9 K, 12 Q, 6 R)
				IM (8 K, 13 Q, 5 R)
				GA (11 K, 5 Q, 1 Y)

**Notes.**

a The given amino acid positions in column 1 are according to reference VacA p55 sequence (Gangwer et al., 2007). Colombia sequences were clustered according to the gastric pathology from which they were obtained as follows: G, Gastritis (31 sequences), AG, atrophic gastritis (27 sequences), IM, intestinal metaplasia (26 sequences), GA: gastric adenocarcinoma (17 sequences). A chi-square test of independence was performed on all non-conserved columns from the protein alignment using a *p < 0.05* as a threshold value. The degrees of freedom were assigned according to the equation: number of observed residues at each aligned position −1 multiplied by the number of groups being calculated −1. Those columns that significantly deviated from random were subject to a Person’s chi-square to determine the pair observed groups skewing of the data (Pickett et al., 2013). DF corresponds to degrees of freedom.

The phylogenetic tree of p55 VacA showed three principal clades –one for the m1 type and the others for the m2 and chimeras in Colombian mestizos, respectively, suggesting a possible functional divergence event. The phylogenetic tree showed very short terminal branches, which indicates that mutations were accumulated over a short period of time and that a bottleneck process took place in the region, where only few organisms survived a strong selective pressure that reduced the population. When the branch distances were ignored, the phylogenetic tree showed multiple points of functional diversification between the p55 VacA variants. This diversification could represent functional adaptation to host cell receptors (Gangwer et al., 2010) and/or immune selective pressure ( Ghose et al., 2007).

A previous study showed that the divergence between *vacA* alleles is due to positively selected surface-exposed sites in the p55 cell binding domain (Gangwer et al., 2010). In this study, the different algorithms also detect positive selected sites in the surface of the protein, but the major contribution to the microevolutionary patterns observed for p55 VacA was made by the strong purifying selection. The positive selected sites detected were defined principally as episodic diversifying residues supporting the observed branching scheme of the phylogenetic tree.

Another important force that shaped the microevolution of p55 VacA was recombination and gene conversion. *Helicobacter pylori* can take-up exogenous DNA from the environment (Nedenskov-Sørensen, Bukholm & Bøvre, 1990) and recombine it with an extremely high frequency (Go et al., 1996; Suerbaum et al., 1998). The number of recombination events detected in Colombian p55 VacA suggests that the strains exchanged DNA following a free recombination pattern. This indicates that, in addition to the low nucleotide diversity, the population exhibits a high level of genetic homogeneity. Perhaps, after the initial Spanish colonization, a small initial population of new *H. pylori* subtypes exchanged genetic information by recombination and gene conversion, producing a highly homogeneous strain pool in this country. It has been shown that recombination is more efficient between related strains than unrelated ones (Israel, Lou & Blaser, 2000; Pinto et al., 2005).

The entire positive selected sites and most of the purifying selected ones were located at positions with a high likelihood to be recognized by the host immune system. We propose that immune selection has triggered the diversification of Colombian p55 VacA, but at the same time those diversified alleles have exacerbated the host immune response contributing to the high prevalence of gastric diseases and gastric cancer observed in this geographic area as analysed by the Red Queen model of evolution (Strotz et al., 2018). One of the most important results presented here is the detection of a plausible relation between natural selection and the gastric disease state. Two specific amino acid positions, R261 and L321 according to the reference p55 VacA (Gangwer et al., 2007), both under positive episodic diversifying selection in the Colombian samples revealed significant sequence variations between different disease state groups, which opens new opportunities for the development of early diagnosis strategies specifically addresses to this region from Colombia.

## Conclusions

Taken together, Latin-America represents an “evolutionary laboratory” for *H. pylori*, and it is possible that a new variant of virulence factors such as VacA has been evolving rapidly in this subcontinent. This rapid evolutionary process has been described for example on the AlpA adhesin in the same region (Gutiérrez-Escobar et al., 2018). We assume that 500 years of colonization provided sufficient time to produce new allelic variants for p55 VacA not only in Colombia, and possibly also in other countries of the region. Further studies should investigate this in more detail in *H. pylori* isolates across Latin America.

##  Supplemental Information

10.7717/peerj.6634/supp-1 Table S1 The strain name, the accession code, and the protein sequences used in here are provided as raw data

10.7717/peerj.6634/supp-2 Table S2 Protein sequence alignment of VacA p55 of colombian isolates of H. pylori is provided as a raw data. In the Table S2, the positions 261 and 321 in p55 were highlighted in blue, the positions mentioned in the Table 2 were presented in green and those corresponding to the Table 3 in red.

## Additional Information and Declarations

### Data Availability

The following information was supplied regarding data availability: The raw data are available in the article and Tables S1 and S2. Table S1 contains the protein sequences and accession codes of the proteins and Table S2 contains the protein sequence alignment with the critical amino acids highlighted.
